# Experimental investigations on children’s early structural representation: a view from classifier phrases in Mandarin

**DOI:** 10.3389/fpsyg.2026.1782713

**Published:** 2026-03-11

**Authors:** Shuyan Zhao, Peng Zhou

**Affiliations:** 1 College of Foreign Languages, Beijing University of Technology, Beijing, China; 2 Department of Linguistics, School of International Studies, Zhejiang University, Hangzhou, China

**Keywords:** child language, classifier phrases, hierarchical structure, linear relations, Mandarin Chinese

## Abstract

Linguistic theories divide on whether children’s early representation of language is based on hierarchical structural relations or on linear relations. To shed further light on this debate, the present study investigated Mandarin-speaking children’s choices of classifiers for noun–noun compounds, where the first noun is a modifier noun, and the second noun is a head noun. In these noun–noun compounds, the classifier agrees with the head noun, rather than the linearly closer modifier noun. Two experiments were conducted. Experiment 1 explored whether 4- to 6-year-olds would choose the classifier for the compounds based on linear distance or structural relations. Experiment 2 examined whether children’s choices were influenced by their lexical knowledge of specific classifiers. The results of Experiment 1 show that 4-, 5- and 6-year-olds all chose classifiers that were compatible with head nouns, favoring the structural relation, but the accuracy rate of the 4-year-olds was significantly lower than that of the 5- and 6-year-olds. The findings of Experiment 2 indicate that once the 4-year-olds’ lexical knowledge of specific classifiers was improved, their accuracy rates also significantly improved. The findings provide new evidence for the proposal that children’s early representation of language is based on hierarchical structural relations rather than linear relations.

## 1 Introduction

The field of child language acquisition mainly concerns how children develop a rich and intricate system of linguistic knowledge within just the first few years of life. To account for this process, two major approaches have been proposed, the nativist approach represented by the theory of Universal Grammar (e.g., Chomsky, 1965, 1981, 2005; Chomsky et al., 2019; Crain et al., 2017; Yang et al., 2017) and the empiricist approach represented by the constructivist theory (e.g., Goldberg, 1995, 2003, 2006; Kidd et al., 2018; Lieven, 2016; Tomasello, 2000). The core debate between the two approaches revolved around the question of whether language learning is primarily driven by innate linguistic knowledge children are born with or by the linguistic input children are exposed to after birth.

The nativist view of language acquisition is based on the “poverty of the stimulus” argument, that is, the linguistic knowledge children attain in the first few years of life vastly exceeds the linguistic input they are exposed to (Berwick et al., 2011; Chomsky, 1957, 1965; Crain, 1991; Lidz and Gagliardi, 2015). The discrepancies between children’s linguistic knowledge and their language input were observed in many studies (e.g., Berwick et al., 2011; Crain, 1991; Crain et al., 2010; Crain and Nakayama, 1987; Crain and Pietroski, 2002; Hornstein and Lightfoot, 1981; Leddon and Lidz, 2006; Legate and Yang, 2002; Li and Zhou, 2020; Lidz et al., 2003; Su et al., 2012; Sugisaki and Murasugi, 2015), which led the nativists to propose that children are born with some abstract linguistic knowledge that guides their language learning.

In contrast, the empiricist approach of language acquisition, in particular the one represented by the constructivist theory, denies the existence of inborn linguistic knowledge and emphasizes that children learn language by witnessing language in use in linguistic environments and by relying on domain-general learning mechanisms, such as imitation, analogy, and distributional analysis (e.g., Goldberg, 1995, 2006; Lieven and Tomasello, 2008; Saxton, 2010; Tomasello, 2000).

**Figure 1:**
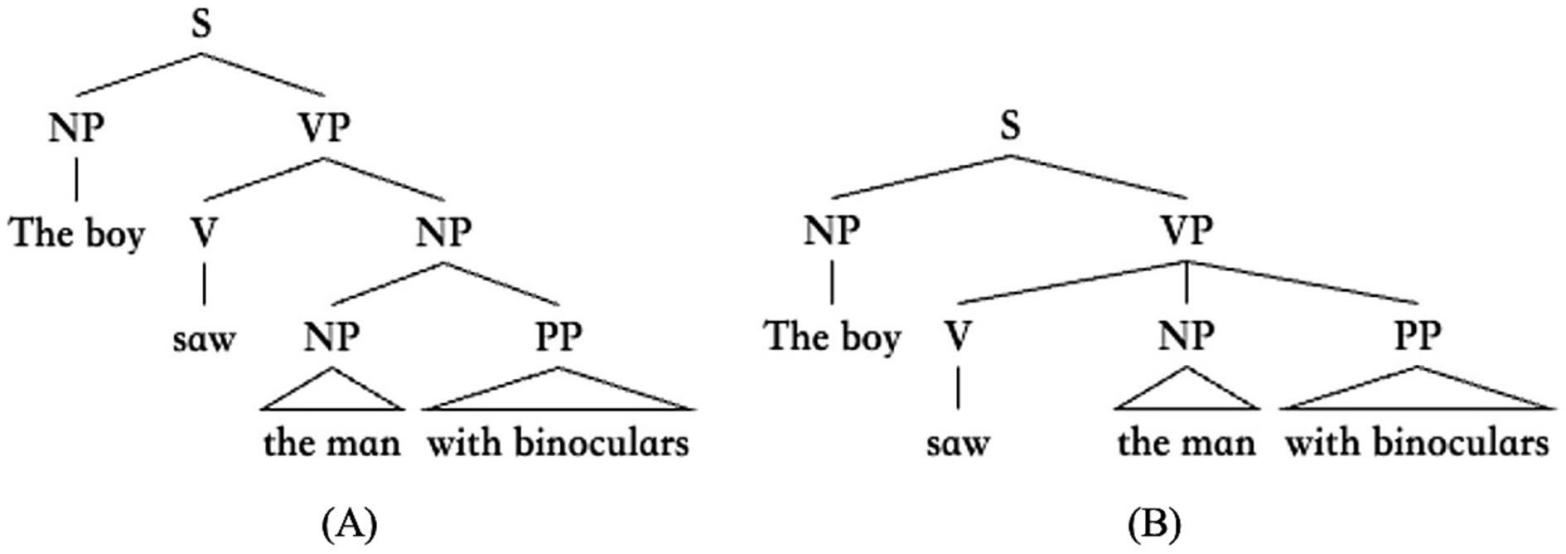
Two hierarchical structures for the sentence “The boy saw the man with binoculars.” **(A)** represents the meaning that the boy saw the man that had binoculars, and **(B)** represents the meaning that the boy saw the man using binoculars.

A fundamental divergence between the two approaches is how language is represented in young speakers’ mind, specifically, whether or not language is represented in the form of abstract hierarchical structure (Everaert et al., 2015; Lidz and Gagliardi, 2015). On the theory of Universal Grammar, language is hierarchically structured, and the meanings that can be assigned to linguistic units are dependent on the abstract structural relations. Consider the example in (1) by Berwick et al. (2011). Sentence (1) is ambiguous depending on which element the prepositional phrase “with binoculars” modifies. When “with binoculars” modifies the object noun phrase “the man,” the sentence means that the boy saw the man that had binoculars, but when “with binoculars” modifies the main verb “saw,” the sentence means that the boy saw the man using binoculars. The ambiguity of word strings like (1) can be well explained if, unlike the linear sequence on the surface, sentences are hierarchically structured, and the two interpretations of (1) are derived from two distinct structures as shown in Figures 1A,B, respectively.

(1) The boy saw the man with binoculars.

A key proposal on this theory is that the hierarchical representation of language emerges early in child grammar (e.g., Crain and Nakayama, 1987; Crain and Thornton, 1998; Everaert et al., 2015; Gleitman, 1990; Lidz, 2010; Wexler, 1990; Yang et al., 2017). Strong evidence for this proposal comes from a classic experimental study by Crain and Nakayama (1987), which investigated whether young children apply structure-dependent or structure-independent rules when formulating yes-no questions. A structure-dependent rule is based upon the hierarchical structure of word sequences, whereas a structure-independent rule relies on linear order and makes reference to linear notations like *first*, *second*, *initial*, or *closest*. To test which rules young children followed, Crain and Nakayama (1987) designed an elicited-production task in which 3- to 5-year-old English-speaking children were prompted to ask Jabba the Hutt particular questions, and the prompt structures were like *Ask Jabba if the man who is watching Mickey Mouse is happy*, which contained an embedded question with two auxiliary verbs. The findings were that most of the children’s yes/no questions were formed by moving the auxiliary verb *is* in the main clause to the front of the embedded sentence, e.g., *Is the man who is watching Mickey Mouse happy?*, adhering to the structure-dependent rule. The children did produce some ungrammatical sentences, but none of them applied structure-independent rules (e.g., moving the *first* auxiliary verb to the front of the embedded sentence) and generated ungrammatical expressions like *Is the man who watching Mickey Mouse is happy?. The findings are strong evidence for the hierarchical representation of language in young children.

By contrast, the constructivist theory focuses on the non-hierarchical aspects of language (Goldberg, 1995, 2003, 2006; Lieven et al., 2009; Lieven and Tomasello, 2008; Tomasello, 2000, 2003). On this theory, language is an inventory of constructions, each of which has particular communicative functions and children’s early utterances stem from the memorization and imitation of concrete construction forms along with their communicative functions in the contexts; the abstract linguistic knowledge, including syntactic categories and structures, is learnt by children in an item-based and piecemeal manner on the basis of input (Goldberg, 2003; Tomasello, 2000, 2003). Consider an example from Tomasello (2003). Children might hear a number of utterances like *I push it*, *I throw it*, *I hit it* from the surrounding environment. These lexically similar expressions vary in only one element and children might learn from the linguistic context that all of these forms are uttered associated with the meaning of the speaker performing some action on an object. Thus, children are likely to generalize across these utterances with similar lexical items and shared meanings and obtain a lexically specific/partially abstract schema *I ACTION it* with the slot of *ACTION* available for various words. Next, children might analogize across similar schemas like *I ACTION it*, *He ACTION it* and *She ACTION it* and draw a more abstract schema *X ACTION it* and finally reach an adult-like fully abstract construction *X ACTION Y*. Notably, on the constructivist approach, children have to rely on the co-occurrence of linear word sequences, in contrast to the theory of Universal Grammar that children’s early language is organized in hierarchical structure.

To shed further light on whether or not children’s early representation of language is built upon hierarchical structure, the present study takes advantage of the typological features of Mandarin Chinese. More specifically, we use the classifier-head relation in Mandarin noun-noun compounds as a window to see whether children’s choice of classifiers depends on hierarchical or linear relations. Before presenting our experimental studies, we first briefly introduce the classifier system and the classifier-head agreement relation of noun-noun compounds in Mandarin, and then we present prior research on Mandarin-speaking children’s acquisition of classifiers.

## 2 Classifiers of mandarin

Mandarin is a classifier language that features a rich classifier system (Chao, 1968; Erbaugh, 2006; Huang et al., 2008; Tang, 1990; Zhu, 1982, among many other). Nouns do not have singular-plural suffixes to mark countability, and both count and mass nouns require the insertion of a classifier between numbers and nouns in order to be counted, as shown in (2a) and (2b).^1^

(2) a. yi/san ge ren

one/three CL person

‘one person/three people’

yi/san ben shu

one/three CL book

‘one/three book(s)’

b. yi/san ping shui

one/three CL water

‘one/three bottle(s) of water’

yi/san wan tang

one/three CL soup

‘one/three bowl(s) of soup’

Due to the lack of a number morphology system, there is not a syntactic way of encoding the distinction between count and mass nouns in Mandarin. However, some researchers claim that the count/mass distinction in Mandarin is encoded at the level of classifiers (Cheng and Sybesma, 1998, 1999; Doetjes, 1996). According to them, Mandarin classifiers can be roughly divided into two types (Cheng and Sybesma, 1998, 1999; Chien et al., 2003; Tai, 1994; Zhang, 2007). The first type only combines with nouns that come naturally in discrete and countable units by which they can be counted, namely count nouns, like *ren* ‘person’ and *shu* ‘book’ in (2a). When combining with these nouns, the first type of classifiers does not create units; they simply name the natural units of the nouns. This type of classifiers is often referred to as count classifiers or sortal classifiers. The second type of classifiers is like measure words in English. When combining with nouns, this type of classifiers creates a unit by which the following nouns can be counted or measured. For nouns that do not have a built-in partitioning like the counterparts of English mass nouns, e.g., *shui* ‘water’ and *tang* ‘soup’ in (2b), they must combine with this type of classifier to build a measurable unit. This type of classifiers is often referred to as mass classifiers/massifiers or mensural classifiers.

A crucial difference between count and mass classifiers is the relationship with the nouns they combine. Count classifiers usually form a relatively fixed and rigid collocation with nouns, whereas the connection between mass classifiers and the following nouns is more temporary and contingent (Li et al., 2010). For example, the specific classifier that can co-occur with *shu* ‘book’ is *ben*, which only collocates with nouns that come in natural volume, like *zazhi* ‘magazine’ and *bijiben* ‘notebook’; the specific classifier that can co-occur with *zhuozi* ‘table’ is *zhang*, which only collocates with nouns with two-dimensional, flat and extended surface including *chuang* ‘bed’, *baozhi* ‘newspaper’ and *youpiao* ‘stamp’. As illustrated by the examples, the common count classifiers in Mandarin categorize a set of nouns with similar characteristics and denote the shape, size, animacy, and function features of the nouns with which they collocate, and the correct choice of specific classifiers requires the generalization of the features carried by the classifiers and the categorization of real-word entities, as well as rote memorization (Fang, 1985; Huang and He, 2025; Li et al., 2010; Tai, 1994).

By comparison, the relationship between mass classifiers and nouns is relatively loose. Many common mass classifiers in Mandarin are derived from nouns, and when they function as classifiers, they can collocate with almost any noun that is compatible with the artificial unit created by the classifier. Thus, on the one hand, the mass classifier *ping* ‘bottle’ can combine with any noun that can be measured by it, including but not limited to *shui* ‘water’, *nai* ‘milk’ and even count nouns like *yingbi* ‘coin’; on the other hand, the mass noun *shui* ‘water’ can collocate with a variety of mass classifiers, like *ping* ‘bottle’, *wan* ‘bowl’, *tong* ‘bucket’, *hu* ‘kettle’, etc.

In summary, a classifier is obligatory between numerals and nouns for counting in Mandarin, and the choice of specific classifiers is dependent on the characteristics of the following nouns. The dependence between classifiers and simple nouns, like *shu* ‘book’ and *shui* ‘water’, is quite straightforward. However, Mandarin possesses a considerable number of compound nouns, especially noun-noun compounds, whose relation with classifiers is more complex. In the following section, we briefly introduce noun-noun compounds in Mandarin and explain how the agreement between classifiers and noun-noun compounds offers a unique diagnostic opportunity for the hierarchy versus linearity debate.

## 3 Classifier-head agreement of mandarin noun-noun compounds

In Mandarin, compounds make up a very important part of its lexicon, and neologisms are mostly created based on compounding rules (Deng, 2021; Dong, 2016). One of the major discussions on compounds is the position of the head noun. Concerning noun-noun compounds (hereafter N1N2 compounds) in Mandarin, they are mostly endocentric with head on the right, which are often referred to as “head-final” (Deng, 2023; Dong, 2016; Gu and Shen, 2001).

A notable feature is that when counting the “head-final” N1N2 compounds, we need to adopt a classifier that agrees with the head noun as the classifier for the compound, even though the classifier is linearly closer to the noun on the left than the head noun on the right. As illustrated in (3a) and (3b), the simple noun *chuang* ‘bed’ is usually preceded by the classifier *zhang*, a specific classifier for countable nouns with flat and extended surface, and *tui* ‘leg’ is typically paired with the classifier *tiao,* a specific classifier for countable nouns in the long, thin, and flexible shape. *Chuang* ‘bed’ and *tui* ‘leg’ can be combined to form a N1N2 compound *chuang-tui* ‘bed post’, with the right-sided *tui* ‘leg’ bearing the core meaning and serving as the head noun, and the left-sided *chuang* ‘bed’ serving as the modifier noun. Thus, when we count the compound, the preceding classifier should be *tiao*, which is compatible with the head *tui* ‘leg’, rather than *zhang* for *chuang* ‘bed’, as shown by the contrast between (3c) and (3d).

(3) a. yi zhang chuang

one CL bed

‘a bed’

b. yi tiao tui

one CL leg

‘a leg’

c. yi **tiao** chuang-**tui**

one CL(for leg) bed-leg

‘a bedpost’

d. *yi **zhang chuang**-tui

one CL(for bed) bed-leg

Note that there is a competition between hierarchical structure and linear distance in the selection of classifiers for the modifier-head N1N2 compounds like *chuang-tui* ‘bed post’, namely, even though N1 is linearly closer to the position of the classifier than N2, the choice of the classifier for the entire compound conforms to the structural rule (i.e., it relies on the remote N2, not on the proximal N1). For children to correctly choose the classifier for the N1N2 compounds, they need to acquire the lexical knowledge of the specific classifiers for N1 and N2, and meanwhile they need to have the structural knowledge that it is the head N2 that determines the preceding classifier.

The current study focuses on the agreement relation between classifiers and N1N2 compounds in Mandarin. By investigating Mandarin-speaking children’s choice of classifiers for the N1N2 compounds, the study intends to provide more empirical evidence as to whether the nominal expressions are represented hierarchically or linearly in the mind of young children. Before moving on to our study, we briefly review prior research on Mandarin-speaking children’s acquisition of classifiers.

## 4 Mandarin-speaking children’s acquisition of classifiers

Earlier studies on Mandarin-speaking children’s acquisition of classifiers asked when and how children acquire the collocation of nouns and the corresponding classifiers (Fang, 1985; Hao et al., 2021; Ying et al., 1983). Ying et al. (1983) and Fang (1985) conducted picture-naming tasks to elicit 4- to 7-year-old Mandarin-speaking children’s production of classifiers and found that even the youngest children adopted the *Num-Cl-N* construction and did not omit or misorder classifiers in counting, which was consistent with the finding of Erbaugh (1986, 2006). However, even though children in the studies never omitted classifiers, they had difficulty in producing the appropriate classifiers for common nouns, and both studies indicated that the acquisition of different classifiers proceeded along distinct timelines [see also Erbaugh (2006)]. According to Ying et al. (1983), the general classifier *ge* is the first classifier acquired by children, followed by the classifier *zhi* that was used to measure small animals; four-year-old children could correctly use *ge* when counting people and some other nouns that are only compatible with *ge*, but they also overuse *ge* in the place of more appropriate specific classifiers and treat it as a general placeholder. According to Erbaugh (2006), specific classifiers like *kuai* (to measure ‘block-like’ entities), *zhang* (to measure flat entities), and *liang* (to measure vehicles), whose usage is restricted and determined by the characteristics of the following nouns, were particularly challenging for children, and children were not able to correctly produce most of these specific classifiers until age 6 or 7, even age 10.

The corpus analysis on Mandarin-speaking children’s classifier production was in line with the experimental findings (e.g., Huang and He, 2025; Li, 2004; Zhou, 2009). Children were found to have already mastered the syntactic properties of classifiers (i.e., the obligatoriness of classifiers in certain syntactic structures) as young as age 2, whereas the acquisition of the semantic properties of specific classifiers, that is the classifier-noun collocations, took several more years and was still incomplete by the end of the preschool year (Erbaugh, 1986; Huang and He, 2025; Zhou, 2009). Huang and He (2025) proposed that children’s semantic acquisition of classifiers followed a lengthy U-shaped, three-stage trajectory—the item-based rote learning stage that is reliant on adult input, the rule-based generalization stage with overextension errors, and the prolonged refinement stage towards full mastery.

Given the protracted period of development in children’s production ability, researchers have conducted experimental studies to investigate how the ability to comprehend classifiers develops (Chien et al., 2003; Fang, 1985; Hao et al., 2021; Hu, 1993; Li et al., 2010). The comprehension experiments adopted the similar design—children were asked to participate in a forced-choice picture or object-selection task in which they need to select from given objects the one that can follow the test classifiers. The results of Fang (1985) showed that most of the 6-year-olds succeeded in using classifiers like *li* (to measure small particles), *zhang* (to measure flat entities), and *tiao* (to measure long and flexible entities) to pick out the target objects and justifying their selection with reasonable statements, but most of the 4-year-olds were not successful. What’s more, the comparison between children’s comprehension and production data revealed that the ability to comprehend classifiers develops earlier than the ability to produce them in Mandarin-speaking children (Chien et al., 2003; Hao et al., 2021; Hu, 1993).

As discussed, the acquisition of Mandarin classifiers relates to the generalization of common features such as shape, size, animacy, function, etc., from associated nouns. Experimental studies have indicated that children’s sensitivity to the shape and size features specified by the classifiers emerges at around age 3 and matures at around age 5, but their awareness of other requirements imposed by the classifiers, like solidity and individuation, develops more slowly (Chien et al., 2003; Huang, 2009, 2019; Huang and Lee, 2009; Li et al., 2008; Li et al., 2010; Sera et al., 2013).

To briefly summarize, prior research has found that Mandarin-speaking children do not omit the obligatory use of classifiers from the onset of the two-word stage around age 2, but the acquisition of the classifier-noun collocations and the shape, size, function, and solidity features encoded in specific classifiers, which relies on the development of children’s generalization ability and the linguistic input, may extend gradually into the school years. Note that these previous studies have primarily focused on classifiers themselves and their collocation with simple nouns, leaving the agreement relation between classifiers and noun-noun compounds underexplored.

To address the gap, the current study investigates children’s selection of classifiers for noun-noun compounds, with the aim of providing further empirical support for the early representation of language in children, as well as the role of linguistic input in child language acquisition. More specifically, two experiments were conducted to examine how 4- to 6-year-old Mandarin-speakers selected the classifiers for the modifier-head N1N2 compounds (i.e., was it based on the modifier N1 or the head N2 of the compounds?), and whether children’s selections were influenced by their lexical knowledge of specific classifiers.

## 5 Experiment 1

### 5.1 Participants

Sixty Mandarin-speaking 4-year-olds (28 girls, age range = 4;3–4;11, mean age = 4;8), 58 5-year-olds (28 girls, age range = 5;0–5;11, mean age = 5;7), and 60 6-year-olds (25 girls, age range = 6;0–6;10, mean age = 6;5) participated in the experiment, and they were all recruited from a kindergarten in Beijing. All the participants had no reported history of speech, hearing, or language disorders.

### 5.2 Materials and design

Twelve common modifier-head N1N2 compounds in Mandarin were selected as the test materials. The 12 compounds and the count classifiers compatible with N1 and N2 of each compound are listed in Table 1. In order to differentiate between children’s choices, the N1 and N2 of all the selected compound nouns take different classifiers, which are named as Cl1 and Cl2, respectively. In addition, given that Mandarin mass nouns can take a number of different mass classifiers, the current experiment only included N1N2 compounds for which both N1 and N2 are count nouns and have a relatively fixed collocation with the corresponding count classifiers.

**Table 1:** N1N2 compounds employed in Experiment 1 and the corresponding count classifiers for N1 and N2 of each compound.

N1N2 compound (object part)	Classifier for N1 (Cl1)	Classifier for N2 (Cl2)	N1N2 compound (body part)	Classifier for N1 (Cl1)	Classifier for N2 (Cl2)
*chuang-tui* ‘bed post’	zhang	tiao	*xiang-ya* ‘ivory’	tou	gen
*che-men* ‘car door’	liang	shan	*ma-wei* ‘horse tail’	pi	tiao
*chuan-fan* ‘ship sail’	tiao	zhang	*ji-mao* ‘chicken feather’	zhi	gen
*hua-ban* ‘petal’	duo	pian	*lang-pi* ‘wolf skin’	zhi	zhang
*cai-ye* ‘cabbage leaf’	ke	pian	*yu-lin* ‘fish scale’	tiao	pian
*shu-zhi* ‘tree branch’	ke	gen	*niu-jiao* ‘ox horn’	tou	gen

All the 12 compounds follow the “whole-part” format, which is a highly productive pattern for constructing modifier-head N1N2 compounds in Mandarin (Chao, 1968; Dong, 2009, 2016). N1 refers to the whole and serves as the modifier, and N2 refers to the part and serves as the head. Consider the N1N2 compound *xiang-ya* ‘ivory’, for example. N1 *xiang* ‘elephant’ is the modifier, N2 *ya* ‘tooth’ is the head, and *xiang-ya* ‘ivory’ is a modifier-head noun-noun compound referring to ‘tooth of elephant’.

In the 12 compounds, six were object parts of inanimate objects, and six were body parts of animals. This manipulation rendered the animacy of N1 different in the two conditions. The inclusion of both inanimate objects and animate animals was to examine whether the animacy of the modifier noun would influence children’s performance.

**Figure 2:**
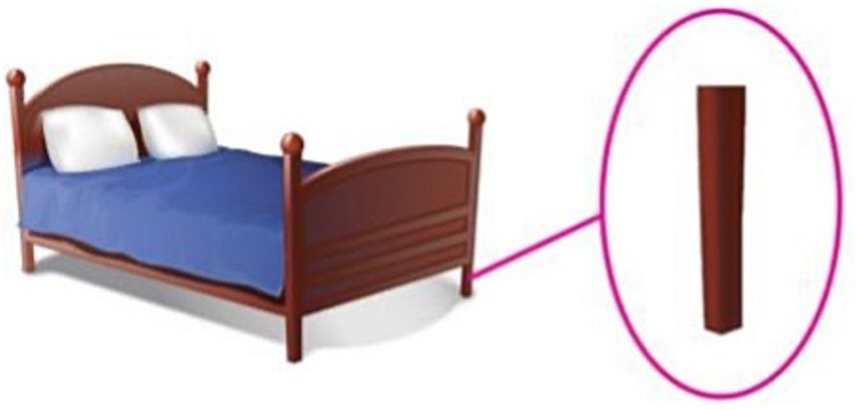
The test picture for the compound *chuang-tui* ‘bed post’. The entity corresponding to N1 (*chuang* ‘bed’) is placed on the left and the entity corresponding to the N1N2 compound (*chuang-tui* ‘bedpost’) is zoomed in and circled on the right.

**Figure 3:**
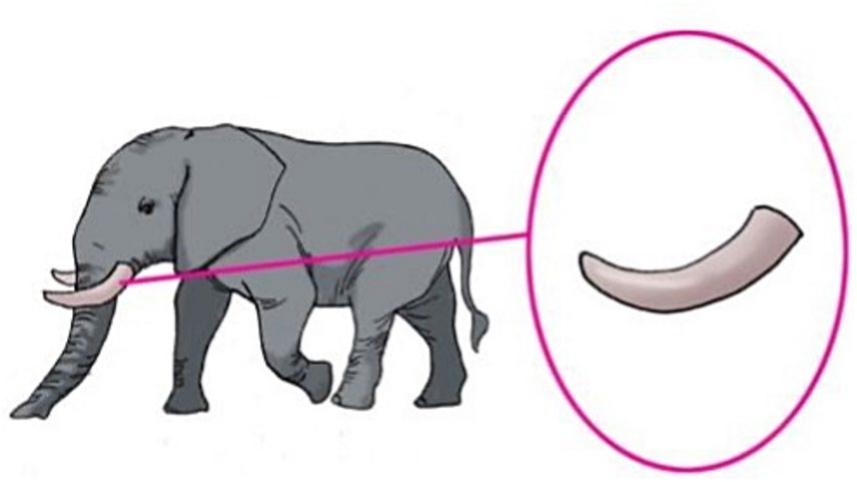
The test picture for the compound *xiang-ya* ‘ivory’. The entity corresponding to N1 (*xiang* ‘elephant’) is placed on the left and the entity corresponding to the N1N2 compound (*xiang-ya* ‘ivory’) is zoomed in and circled on the right.

To depict the 12 compounds, 12 test pictures were constructed. In each picture, the entity corresponding to N1 was presented on the left, and the entity corresponding to the N1N2 compound is zoomed in on the right. An example test picture for the “object-object part” compound *chuang-tui* ‘bed post’ is given in Figure 2, and an example test picture for the “animal-body part” compound *xiang-ya* ‘ivory’ is given in Figure 3. In Figure 2, a bed is presented on the left, and one of the bedposts is zoomed in and circled on the right; in Figure 3, an elephant is presented on the left, and one of the ivories is zoomed in and circled on the right. All twelve test pictures were created in the same manner.

### 5.3 Procedure

The participants were tested using a forced-choice judgment task. They were invited to play a game with two puppets, a kitten and a bunny, and were instructed that the two puppets were learning Mandarin and they would sometimes make language mistakes. In the task, the two puppets had a Mandarin competition by describing the zoomed-in part of the test pictures, and the participants were invited to be the judge to decide which of them better described the picture. More specifically, in the task the experimenter presented the test pictures to the puppets and the participants one at a time. Every time the kitten and the bunny saw a picture as in Figures 2, 3, one of the puppets pointed to the zoomed-in part on the right and uttered the test sentence *zhe shi yi-Cl1 N1N2* ‘this is one-Cl1 N1N2’ with the *N1-*compatible classifier *Cl1* as the classifier for the compound (e.g., *zhe shi yi-**zhang chuang**-tui* for Figure 2), the other puppet uttered the test sentence *zhe shi yi-Cl2 N1N2* ‘this is one-Cl2 N1N2’ with the *N2-*compatible classifier *Cl2* (e.g., *zhe shi yi-**tiao** chuang-**tui*** for Figure 2). In the task, the kitten always spoke first followed by the bunny, but the two types of sentences were counterbalanced across the two puppets, i.e., each puppet produced correct structures on six of the 12 test trials. After the two puppets finished their answers, the participants were asked to judge which of the two puppets better described the zoomed-in part of the picture. It was made clear to the participants that for each picture they could only choose one puppet.

In addition to the 12 test trials, 12 control trials were also created to see whether the participants understood N1N2 compounds. As on the test trials, on the control trials the two puppets competed to describe pictures and the participants were invited to be the judge. The 12 pictures were the same as the pictures of the test trials, but the structures used by the puppets were different. On the control trials, one puppet pointed to the zoomed-in part of the picture and described it using the correct name N1N2 in the bare form, and the other produced an incorrect name in the bare form. The incorrect name was also a N1N2 compound, but differed with the correct one in the modifier N1 on six control trials, and in the head N2 on the other six control trials. For instance, when shown Figure 2, the kitten said “*zhe shi **chuang**-tui*” ‘this is **bed**post’, and the bunny said “*zhe shi **zhuo**-tui*” ‘this is **table** leg’. The kitten always spoke first followed by the bunny, but the correct and incorrect names were counterbalanced across the two puppets, that is, on six control trials the correct name was produced by the kitten, and on the other six the correct name was produced by the bunny. The control trials were included to make sure that the participants were familiar with all the N1N2 compounds employed in the experiment and had knowledge of how the modifier noun and head noun combine to contribute to the meaning of the compound.

The participants were tested individually in a quiet room in the kindergarten. The 24 trials were presented to the participants in a randomized order. The participants’ choices for each trial were recorded by the experimenter on the answer sheet for later analysis.

### 5.4 Results and discussion

Two 4-year-olds did not finish the experiment because they could not concentrate on the task; another two 4-year-olds and three 5-year-olds always hesitated to give their answers and changed their answers from time to time. These 7 participants were excluded from the data analysis. The other participants performed close to ceiling on the control trials, suggesting that they had knowledge of the N1N2 compounds. Therefore, their data were included in the analysis. The final sample included 56 4-year-olds, 55 5-year-olds, and 60 6-year-olds. In the analysis, we focused on children’s performance on the test trials, where they were asked to choose a better description between *zhe shi yi-Cl1 N1N2* ‘this is one-Cl1 N1N2’ and *zhe shi yi-Cl2 N1N2* ‘this is one-Cl2 N1N2’. The dependent measure was children’s proportion of correctly choosing the N2-compatible classifiers.

The results showed that the 4-year-olds chose the correct description where the classifier matched the head noun 65.28% of the time, and the 5-year-olds and the 6-year-olds did so 84.23% and 87.80% of the time, respectively. To evaluate the possible impact of animacy of the modifier noun N1 on children’s performance, proportions of correct responses on the animate (“animal-body part” N1N2 compounds) and inanimate (“object-object part” N1N2 compounds) trials were calculated separately. The 4-year-olds responded correctly to the animate trials 61.67% of the time, and to the inanimate trials 68.89% of the time. The accuracy rates on the animate and inanimate trials were 79.17% and 89.29% for the 5-year-olds, and 87.50% and 88.10% for the 6-year-olds.^2^

To statistically assess the response patterns of the three age groups, generalized linear mixed models (GLMMs) were applied. We used the functions *lmer* from package *lme4* (v1.1–12) (Bates et al., 2015) of the R (v4.5.1) software environment to conduct the fitting process (R Core Team, 2025). The correct and incorrect selections of classifiers were treated as two levels of the dependent variable. Participants’ correct selections in both animate and inanimate conditions were coded as 1 and the incorrect selections were coded as 0. In building the statistical model, the maximal model included *age group* (i.e., 4-, 5-, and 6-year-old), *condition* (i.e., animate and inanimate) and their interaction as fixed effects, with random intercepts and slopes for participants and random intercepts for items. The complexity of the maximal model was then reduced step by step and the simplified model was compared with the more complex ones to see whether the simplified model presented a similar or better fit of the data. If yes, the simplified model was adopted. The final model included age group and test condition as fixed effects, with random intercepts for participants and items (Formula in R: accuracy ~ age + condition + (1|participant) + (1|item)).

The model results revealed a significant main effect of *age group* [*χ*^2^(2) = 22.96, *p* < 0.001], whereas the main effect of *condition* was not significant [*χ*^2^(1) = 3.58, *p* > 0.05]. Both the 5- and 6-year-olds provided significantly more correct responses than the 4-year-olds (5-year-olds versus 4-year-olds: *β* = 1.44, *SE* = 0.40, *z* = 3.64, *p* < 0.001; 6-year-olds versus 4-year-olds: *β* = 1.79, *SE* = 0.41, *z* = 4.39, *p* < 0.001), and no significant difference was observed between the 5- and 6-year-olds (*β* = −0.35, *SE* = 0.43, *z* = −0.83, *p* > 0.05). The model results suggested that the performance of the 5- and 6-year-olds in choosing the correct N2-compatible classifier for the N1N2 compounds was significantly better than that of the 4-year-olds, and the animacy of the modifier noun N1 did not have a significant influence on the participants’ choices.

To better understand the nature of the 4-year-olds’ errors, we did an error analysis. The major findings were that children’s accuracy rates varied across trials. For example, the 4-year-olds chose the incorrect N1-compatible classifiers for the N1N2 compounds *xiang-ya* ‘ivory’ and *chuan-fan* ‘ship sail’ only 20% of the time, but they gave incorrect responses on the trial of *lang-pi* ‘wolfskin’ 50% of the time. But, at the same time, the 4-year-olds exhibited consistently lower performance across all three N1N2 compounds taking *pian* as the N2-compatible classifier, including *hua-ban* ‘petal’, *cai-ye* ‘cabbage leaf’, and *yu-lin* ‘fish scale’.

The 4-year-olds’ variability in performance across trials can be explained by two competing hypotheses. The first hypothesis is that children’s selection of classifiers for N1N2 compounds reflected their abstract structural knowledge of classifier-head dependency, and the variability across trials was due to the lexical acquisition of specific classifiers, namely, their command of different classifiers varied. The second hypothesis is that children’s classifier selection did not require structural knowledge of the modifier-head relation. Instead, the 4-year-olds treated N1N2 compounds as single, unanalyzed lexical chunks, and thus needed to learn the compatible classifiers by rote on an item-by-item basis. On the first hypothesis, children’s performance depends on their knowledge of how target classifiers collocate with the head nouns of the N1N2 compounds, but on the second hypothesis, it is the knowledge of how target classifiers collocate with N1N2 compounds as a whole that determines children’s choices.

Notably, the 4-year-olds’ consistent difficulty with all compounds taking *pian* as the head-compatible classifier seems to support the first hypothesis because it hints at the connection between the classifiers and the head nouns, which mainly relies on the rule-based generalization of the common features shared by all the nouns that follow a specific classifier, rather than an independent learning for each classifier-compound collocation. In order to further identify the underlying causes of the relatively lower performance of the 4-year-olds in Experiment 1 and empirically adjudicate between the two competing hypotheses, we conducted a second experiment, a training-based experiment. In Experiment 2, Mandarin-speaking 4-year-olds were taught the classifiers that were compatible with the modifier and the head nouns, respectively, before they were asked to select classifiers for the N1N2 compounds. If the first hypothesis is true, children would be expected to show a higher accuracy rate than in Experiment 1 after training. If the second hypothesis is true, no significant difference in children’s performance would be expected across the two experiments. The following section presents Experiment 2.

## 6 Experiment 2

### 6.1 Participants

Fifty-six Mandarin-speaking 4-year-olds (26 girls, age range = 4; 2–4; 11, mean age = 4; 7) participated in the experiment, and they were all recruited from a kindergarten in Beijing. All the participants had no reported history of speech, hearing, or language disorders. None of the participants had participated in Experiment 1.

### 6.2 Materials and design

Eight modifier-head N1N2 compounds were selected from Experiment 1 and were used as the test materials, including *chuang-tui* ‘bed post’, *che-men* ‘car door’, *chuan-fan* ‘ship sail’, *shu-zhi* ‘tree branch’, *xiang-ya* ‘ivory’, *ma-wei* ‘horse tail’, *ji-mao* ‘chicken feather’, and *niu-jiao* ‘ox horn’. As in Experiment 1, the first four compounds were parts of inanimate objects, and the latter four were body parts of animals.

**Figure 4:**
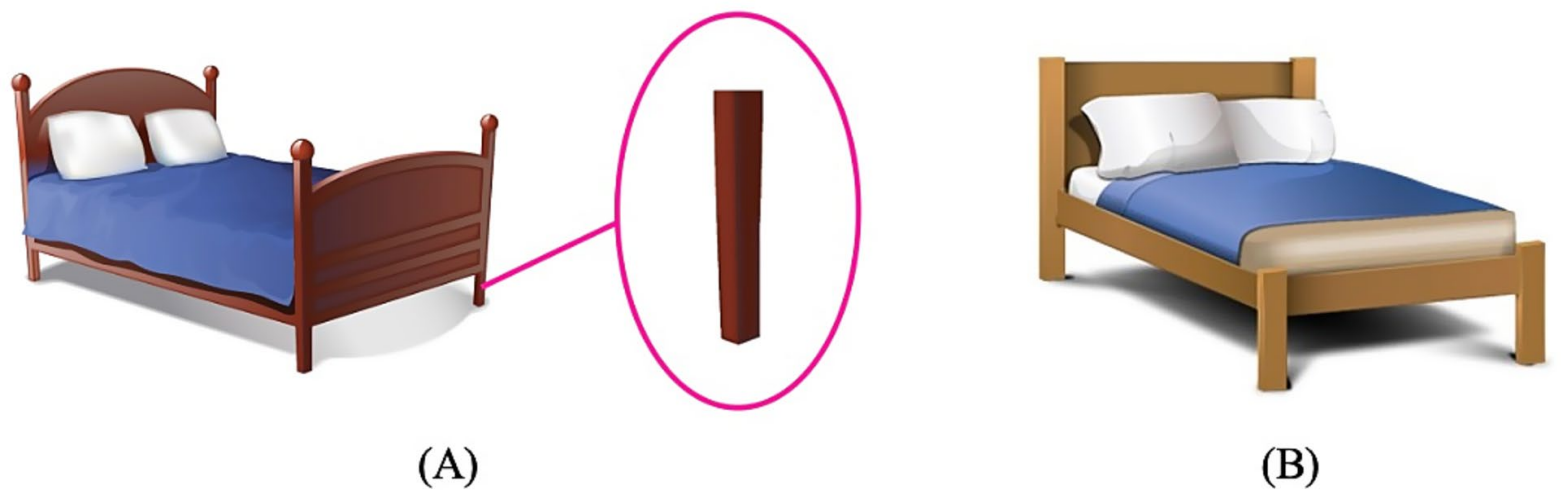
The training picture **(A)** and the test picture **(B)** for the compound *chuang-tui* ‘bed post’. **(A)** presents the entity corresponding to N1 (*chuang* ‘bed’) on the left and the entity corresponding to the N1N2 compound (*chuang-tui* ‘bedpost’) on the right, and **(B)** depicts a new entity corresponding to N1 (*chuang* ‘bed’) that is different from the one presented in **(A)**.

**Figure 5:**
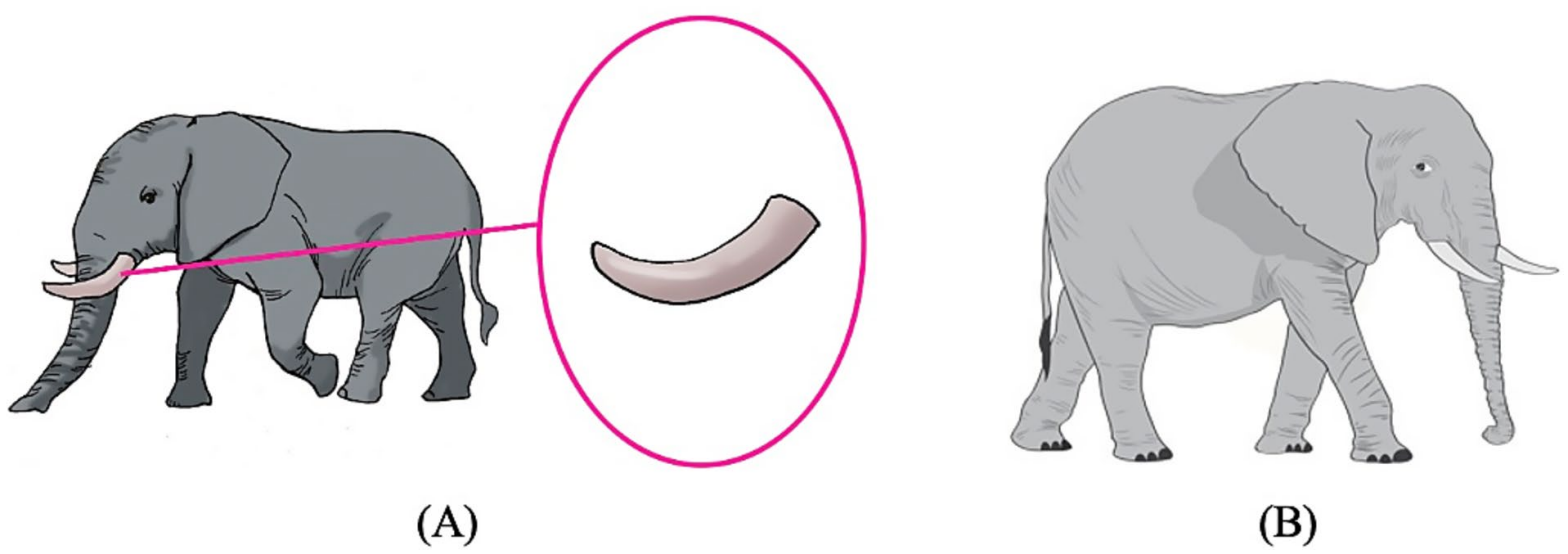
The training picture **(A)** and the test picture **(B)** for the compound *xiang-ya* ‘ivory’. **(A)** presents the entity corresponding to N1 (*xiang* ‘elephant’) on the left and the entity corresponding to the N1N2 compound (*xiang-ya* ‘ivory’) on the right, and **(B)** depicts a new entity corresponding to N1 (*xiang* ‘elephant’) that is different from the one presented in **(A)**.

Two pictures were constructed for each of the eight N1N2 compounds, a *training* picture and a *test* picture. The *training* pictures were the same as the test pictures in Experiment 1 with the entity corresponding to N1 presented on the left and the entity corresponding to the N1N2 compound zoomed in and circled on the right. The *test* pictures depicted a new entity corresponding to N1 that was different from the one presented in the *training* pictures. For illustration purposes, the training and test pictures for the test compounds *chuang-tui* ‘bed post’ and *xiang-ya* ‘ivory’ are given in Figures 4, 5 respectively. Figures 4A, 5A are the same as the test pictures of Experiment 1, and Figures 4B, 5B present a new picture of the entire bed/elephant. In the test pictures, the number of object/animal parts corresponding to the referents of the N1N2 compounds was made easily identifiable to the participants, because in the task the participants were asked to count the number of N1N2. For instance, we can see clearly that there are three bed posts in Figure 4B and two ivories in Figure 5B.

### 6.3 Procedure

The participants were tested using a training-based judgement task. They were invited to play a game with two puppets, a bear and a bunny, and were instructed by the experimenter that the two puppets came from the Animal Planet and spoke their own language. In the task, the two puppets invited the 4-year-olds to learn the language of the Animal Planet with them.

Each test trial consisted of a training phase followed by a test phase. The training phase was designed to teach the participants the corresponding classifiers for N1 and N2, and the test phase was to examine which classifier of the two the participants would select as the classifiers for the N1N2 compounds after training. In the training phase, the participants were shown the training pictures of the N1N2 compounds. One of the puppets pointed at the entity corresponding to N1 on the left side of the training pictures and asked the children “What is it?.” After the children produced an answer *x*, the puppet would immediately follow up by asking “How many *x* are there in the picture?.” Note that the original questions were presented in Mandarin, and the motivation for including the follow-up question was to prompt children to produce the *Num-Cl-N* structure for the modifier noun N1. If the participants produced the correct classifier Cl1, the puppet would say “On our Animal Planet we speak in the same way” and then repeated what the children had just produced by saying “We also say *Num-Cl1-N1*”; but if the participants produced an inappropriate classifier for N1, the puppet would correct them by saying “On our Animal Planet, we do not say this. We say *Num-Cl1-N1*. Could you please learn with me?,” and then the puppet asked the children to repeat the correct collocation until they could produce the form by themselves.

After this, the second puppet pointed at the zoomed-in part corresponding to the N1N2 compound on the right side of the training pictures and asked the children “What is it?.” After the children gave a name *y*, the puppet would ask “How many *y* are there in the picture?.” For the first question, the expected answer was the head noun N2 alone. If children produced an answer with the modifier N1, for example in the form of *N1N2* or *N1-de-N2*, the puppet would confirm their answer but only emphasize N2 by saying “Yes, this is N2” and asked “How many N2 are there in the picture?” as the second question to prompt children to produce the *Num-Cl-N* structure for the head noun N2. For the second question, if the children gave the expected answer *Num-Cl2-N2* with the appropriate classifier Cl2, the puppet would say “On the Animal Planet we speak in the same way” and then repeated what the children had produced by saying “We also say *Num-Cl2-N2*”; but if the children produced an inappropriate classifier other than Cl2 for N2, the puppet would correct them by saying “On our Animal Planet, we do not say this. We say *Num-Cl2-N2*. Could you please learn with me?,” and then the puppet asked the children to repeat the correct collocation until they could produce the form by themselves. Note that in the training phase, only the classifiers compatible with the components of the compound (e.g., *zhang* for *chuang* ‘bed’, *tiao* for *tui* ‘leg’) were taught, never the compound itself.

In the test phase, the participants were presented with the corresponding test pictures, each depicting a new instantiation of N1 that was different from the one in the training pictures. The two puppets asked “Could you tell me how many N1N2 are there in this picture using the language of the Animal Planet I have just taught you? Is it *Num-Cl1-N1N2 or *Num-Cl2-N1N2*?” Since in the training phase the children were taught the N1- and N2-compatible classifiers, thus in the test phrase when asked to count the number of N1N2, they should have the lexical knowledge that was required in order to select between Cl1 and Cl2 the classifier compatible with the N1N2 compounds.

The participants were tested individually in a quiet room in the kindergarten, and were presented with the 8 test trials in random order. In addition, we counterbalanced which of the puppets (the bunny or the bear) spoke first in the training phase as well as whether the correct *Num-Cl2-N1N2* or the incorrect *Num-Cl1-N1N2 utterances were presented first in the test phase. The participants’ responses were recorded by the experimenter for later analysis.

### 6.4 Results and discussion

The data from two children were excluded, because one child could not concentrate on the task, and one always hesitated to give his answers and changed his mind from time to time. The other 54 4-year-olds all successfully completed the task and thus were included in the final analysis. The dependent measure was children’s proportion of correctly choosing the N2-compatible classifiers.

The results showed that in this task the 4-year-olds chose *Num-Cl2-N1N2* over *Num-Cl1-N1N2 84.48% of the time. In order to explore whether the training on the lexical and collocational knowledge of specific classifiers had an impact on the participants’ choice of the classifiers for the N1N2 compounds, we statistically compared the 4-year-olds’ performance in the current experiment with their performance in Experiment 1 on the eight test trials that were included in both experiments.

Again, GLMMs were employed to make the comparison. We conducted the same fitting process as in Experiment 1 via functions *lmer* from package *lme4* (v1.1–12) (Bates et al., 2015) of the R (v4.5.1) software environment (R Core Team, 2025). The best-fitting model included *group* (i.e., the 4-year-olds in Experiment 1 versus those in Experiment 2) and *condition* (i.e., animate versus inanimate) as fixed effects, with random intercept for participants and items (Formula in R: accuracy ~ group + condition + (1|participant) + (1|item)).

The results revealed that *group* is a reliable predictor of the participants’ choices of the correct classifiers, whereas *condition* is not. The 4-year-olds in Experiment 2 provided significantly more correct responses than the 4-year-olds in Experiment 1 (*β* = 1.11, *SE* = 0.27, *z* = 4.08, *p* < 0.001).

To sum up, after being trained on the lexical knowledge of the specific classifiers compatible with N1 and N2 in the N1N2 compounds, the Mandarin-speaking 4-year-olds correctly chose Cl2 as the classifier for the N1N2 compounds significantly more often than those who were not trained on the knowledge. The finding, in particular, the significantly higher accuracy rate of the 4-year-olds in the current experiment than those in Experiment 1 lends support to the first hypothesis, indicating that children did represent the nominal on the basis of the hierarchical structure, and their performance was subject to the lexical knowledge of specific classifiers at the same time.

## 7 General discussion

In the current study, we sought to provide new data that could shed light on whether or not children’s early representation of language is built upon hierarchical structure. We took advantage of the typological features of Mandarin, that is, Mandarin is a classifier language in which all nouns require the insertion of a classifier between numerals and nouns in order to be counted. More specifically, two experiments were conducted to investigate which classifier (i.e., the modifier-compatible classifier Cl1 or the head-compatible classifier Cl2) 4- to 6-year-old Mandarin-speaking children would select as the classifier for the modifier-head N1N2 compounds. The idea is that if children represent the nominal expression on the basis of linear sequence, they were expected to exhibit a preference for Cl1 as the classifier for the N1N2 compound since the modifier noun N1 is linearly closer to Cl1 than the head noun N2. By contrast, if they represent the nominals hierarchically, they would consistently choose Cl2 instead because N2 is the head noun that determines the syntactic category and core meaning of the N1N2 compound.

The results of Experiment 1 indicated that the 4-, 5- and 6-year-olds all favored the classifier paired with the head noun over the one that matched the modifier noun as the classifier for the N1N2 compound, but the accuracy rate of the 4-year-olds was significantly lower than that of the 5- and 6-year-olds. A further analysis on children’s incorrect choices revealed that the performance of the 4-year-olds varied across trials, which leads to two hypotheses. The first hypothesis is that the 4-year-olds’ choice was based on the classifier-head dependency structure, but their collocational knowledge of the classifiers and the head nouns was not in place; the second is that they treated N1N2 compounds as unanalyzed chunks, and had not fully acquired the compatible classifiers. Experiment 2 was conducted to adjudicate between the two hypotheses. The results showed that after training on the corresponding classifiers for the head and modifier nouns respectively before the test, the 4-year-olds made significantly more correct responses than those without training in Experiment 1, supporting the first hypothesis. Taken together, the findings of the two experiments suggest that Mandarin-speaking 4- to 6-year-olds are able to consistently choose the classifier for the N1N2 compounds on the basis of hierarchical structure rather than linear order, as long as they are equipped with the lexical knowledge of the classifier-head collocations.

At this point, a natural question to ask is: how do children acquire this hierarchical relation between the classifier and the head noun in the N1N2 compounds? One possibility that people can think of is that children use distributional cues in the input to build the connection between the classifier and the head noun, that is, there are sufficient data in the adult input that enables children to discern that the classifier used before the N1N2 compounds should be compatible with the head noun N2 rather than the modifier noun N1. In order to test this possibility, we conducted a corpus study on the child-directed utterances in *Zhou3* corpus from the CHILDES database (MacWhinney, 2000; Zhang and Zhou, 2009). The *Zhou3* corpus recorded the naturalistic conversation between a Mandarin-speaking girl, whose production was traced from 20 months to 65 months, and her parents and grandparents under the context of free play interaction in the kindergarten, including 30 sessions of speech data with 24,009 utterances in total. For the purpose of the current study, we specifically focused on the modifier-head noun-noun compounds in the child-directed speech.

In a survey of 15,561 parental and grandparental utterances in *Zhou3* corpus, 22 instances were found in which the modifier-head N1N2 compounds are preceded by a classifier. However, out of the 22 instances, 16 take the general classifier *ge*, and four of them are presented in (4). The problem with these utterances is that as the general classifier, *ge* is acceptable to collocate with most simple nouns in Mandarin. For instance, the mother in *Zhou 3* corpus spontaneously produced *zhe ge shu* ‘this book’ and *yi ge hua* ‘a flower’ in the conversation with her daughter, even though the nouns *shu* ‘book’ and *hua* ‘flower’ in Mandarin are typically preceded by the specific classifiers *ben* and *duo*, respectively. There are two major observations. First, the large proportion of the general classifier *ge* in the classifier-compound instances further rejects the second hypothesis, because the overuse of *ge* in the linguistic input could not suffice to help children acquire the collocation of specific classifiers and N1N2 compounds as single units. More importantly, the cooccurrence of the general classifier *ge* and the modifier-head N1N2 compounds in the linguistic input could not inform children of whether the preceding classifier is determined by the modifier or the head noun either. In other words, expressions like (4) with the general classifier *ge* are insufficient to provide children with positive evidence of the classifier-head agreement regarding the modifier-head N1N2 compounds.

(4) a. zhe ge xue-ren

this CL snow-man

‘this snowman’

b. hua ge shu-ye

draw CL tree-leaf

‘draw a leaf’

c. yi ge cha-bei

one CL tea-cup

‘one teacup’

d. yi ge qi-qiu

one CL air-ball

‘one balloon’

In addition, there are only 6 instances in which the modifier-head N1N2 compounds co-occur with specific classifiers, which are listed in (5). A close look at these expressions revealed that only the N1N2 compound *shou-qiang* ‘handgun’ in (5a) is similar to the type of compounds we employed in the experiments (i.e., both N1 and N2 are count nouns, and they have a relatively fixed collocation with different count classifiers—the count nouns *shou* ‘hand’ and *qiang* ‘gun’ take the count classifiers *zhi* and *ba* respectively). In all the other cases of (5), either N1 is not a count noun or both N1 and N2 are compatible with the preceding classifier. For example, the modifier *huo* ‘fire’ in (5c) and *qi* ‘steam’ in (5d) are all mass nouns. Thus, the parental and grandparental speech in the corpus contained only one clear example of the agreement relation between the classifier and the head noun of the N1N2 compounds, and such low frequency of occurrence makes it quite unlikely that children learn the hierarchical relation solely based on linguistic input.

(5) a. zhe ba shou-qiang

this CL hand-gun

‘this handgun’

b. yi ba zhi-qiang

one CL paper-gun

‘this paper gun’

c. yi gen huo-chai

one CL fire-firewood

‘a match’

d. ji liang qi-che

several CL steam-vehicle

‘several cars’

e. bei shou tang-shi

recite CL Tang-poem

‘recite a Tang poem’

f. yi zhang tu-zhi

one CL drawing-paper

‘a piece of drawing’

Overall, the corpus analysis revealed that there was little positive evidence in the adults’ utterances attesting to the agreement relation between the classifier and the head noun of the modifier-head N1N2 compounds. However, young Mandarin-speaking children in the experiments already exhibited hierarchy-based knowledge in choosing the head-matched classifier for the N1N2 compounds. The fact that the linguistic input does not suffice for children’s language development (aka the huge discrepancy between children’s linguistic knowledge and their linguistic input) lends support to the nativist view of language acquisition, according to which children’s hierarchical representation of noun-noun compounds in the early grammar might be innately specified (e.g., Berwick et al., 2011; Chomsky, 1957, 1965; Crain, 1991).

But, we also wish to note that linguistic input does have a role to play in children’s acquisition of classifiers. Previous studies have shown that the acquisition of the lexical knowledge of specific classifiers in Mandarin-speaking children extends over a long period of time, and different classifiers are acquired at different ages (e.g., Fang, 1985; Hao, 2019; Hao et al., 2021; Hu, 1993; Huang and He, 2025; Ying et al., 1983; Zhou, 2009). Consistent with prior research, children’s performance in Experiment 1 varied across classifiers, suggesting that their selection of the classifiers is subject to both the structural knowledge of the modifier-head compounds and the lexical knowledge of the classifier-head collocations. In Experiment 2, the significant improvement of the 4-year-olds after training on the classifiers that were compatible with the head and modifier nouns indicated the significance of the linguistic input for classifier acquisition.

The early acquisition of the syntactic structure of nominals and the protracted development of the semantic knowledge of classifiers in Mandarin-speaking children echo a recent finding of a large-scale corpus analysis on classifier acquisition (Huang and He, 2025). Huang and He (2025) observed a dissociative acquisition between the syntactic and semantic aspects of Mandarin classifiers. By showing how children’s syntactic performance is constrained by their semantic knowledge of specific classifiers as well as how the growth in children’s semantic knowledge could enhance their syntactic performance, the current study not only provides evidence for the relative independence of syntactic and semantic development, but also demonstrates how the semantic development is intertwined with syntactic development during child language acquisition.

In summary, our experimental findings provide empirical support for the hierarchy-based representation of language in young children, which aligns with the nativist view of language acquisition. It is worth noting that the nativist approach of language acquisition, though emphasizing the decisive role of *nature*, acknowledges the important role that linguistic input plays in triggering the relevant innate knowledge (e.g., Chomsky, 2005, 2011; Yang et al., 2017). On the theory of Universal Grammar, child language is argued to be the results of the interplay of three factors: domain-specific principles of language, external experience, and domain-general cognitive abilities (Chomsky, 2005, 2011; Yang et al., 2017). Our findings on Mandarin-speaking children’s selection of classifiers for noun-noun compounds reveal that all the three factors, including the domain-specific structural knowledge of noun-noun compounds, the lexical knowledge of specific classifiers from external environment and the domain-general cognitive abilities required to categorize nouns by the characteristics of specific classifiers, contribute to children’s representation of the nominal expressions. Further research is required to investigate how the three factors interact in the process of child language acquisition.

## Data Availability

All the test materials, analysis codes and results for the study are available in Open Science Framework via the link: https://osf.io/z3xsf/overview?view_only=3db01ecfd87442fbb89dd1a7a80c72f1.

## Footnotes

1 As pointed out by one of the reviewers, some researchers argued that Mandarin Chinese adopts some plural markers, like the suffix -*men* (e.g., Jiang, 2017; Li, 1999). But note that -*men* is reserved for animate nouns in Mandarin and the absence of it does not suggest singularity. For the purpose of the current study, we will not go into the details here. Interested readers are referred to the cited references.

2 One of the reviewers raised the question of why the accuracy rate of the 5- and 6-year-olds on the animate trials did not increase with age. One possible explanation might be relevant to children’s difficulty with the collocational knowledge of the test classifiers and head nouns, of which the acquisition requires a protracted period of time (e.g., Erbaugh, 1986; Huang and He, 2025; Zhou, 2009). Further research is required to investigate this possibility.
